# Round the Hospitals

**Published:** 1916-04-15

**Authors:** 


### ROUND THE HOSPITALS

A representative meeting of hospital authorities and workers like that at St. Thomas's Hospital on the 7th instant supplies the observant with much food for thought. Our modern hospitals have grown so much in size and importance in the last thirty years, especially when they have attached to them medical and nursing schools, one or both, that many of them are, in fact, small republics, where the life is so interesting and absorbing as to leave little time for the staff to occupy themselves with outside matters. How many of the most experienced workers until the last few years made it their business to spend a portion of their limited leisure in inspecting other institutions, to become familiar with their system or management, equipment, and so forth? There are to-day well-administered hospitals, the reports of which entitle them, from their quality and interest, to the close study of hospital workers. There are hospitals where the nursing departments are splendidly organised and administered; where the literature and information prepared on the inspiration of an able matron constitute records of permanent value and interest. These publications are wholly apart from the necessary and confidential registers of books which are necessarily kept by the matron, who may naturally preserve them under lock and key. But apart from these working necessities of a large training school, there exist to-day Registers which have been printed, and one at least may be obtained through a bookseller at a small price. These Registers have many uses, not the least of which is the opportunity they afford to the supporters of a hospital to obtain competent nurses through the private staff, and to ascertain beforehand the quality of the training and the character of the work which their nurse or nurses have undergone or done. The College of Nursing will, we hope, one day have a library which will contain a copy of the history of every nurse-training school and of every such Register of its nurses.

The matrons who were present at the meeting to consider the College of Nursing, held at St. Thomas's Hospital on April 7, appeared greatly amused at the assertion made by the medical superintendent of the Shoreditch Poor-Law infirmary that nurses' training is conducted by medical men, continued ripples of laughter making it difficult to hear the contentions of the speaker. Few medical men will deny that the training of nurses rests primarily with matrons and sisters. In the wards all orders reach the pupil-nurse through the ward sister, and, though the lectures on the symptoms and treatment of disease are given by medical men or women, the actual nursing work is taught both in the wards and lecture-room by the matron and her sisters, while the decision as to the subjects taught, the time allotted for study, the organisation of lectures and examinations is in most hospitals one of the most important of the matron's duties. When we hear criticisms as to the manners and competence of the nurses belonging to a training school we cannot remember a single instance of blame being attached to the medical officers. The matron is always blamed for the shortcomings of her staff, and the influences which go to form the character of the pupil nurse are determined by the matron. Certainly the decision that two-thirds of the Council of the College of Nursing are to consist of hospital matrons is a right one.

The question of pensions for nurses disabled from the war is already engaging the attention both of nurses and the public. There is a general feeling abroad that the disabled nurse must, no more than the disabled soldier, be allowed to suffer either from pecuniary anxieties or actual want. The scale of pensions awarded to nurses working under the War Office and the Admiralty will doubtless be augmented on such a scale as to allow each member of the Q.A.I.M.N.S. and the R.N.N.S. to live in reasonable comfort when either ill-health or age prevents her from earning her living. It is to be hoped that the system of providing homes for invalid or aged nurses will not be adopted except for special cases. Most nurses prefer to live in places of their own choosing, probably near a married sister or some other relative, where they can take their share in the family and social life of their own circle. Here they can live in happiness free from the restrictions of institutional life. The building and equipping of homes is an expensive matter, and nurses, who are drawn from all sections of society, do not appear to the best advantage when herded together, nor when obliged to mix with others who may be uncongenial to them. It is often irksome to conform to rules which, though necessary in a community, are distasteful to women who have earned the right to choose their own mode of life.

The Contracts Committee of the St. Pancras Guardians decided to supply the patients at the North and South Infirmaries with a good "butter substitute" in place of butter. At the Board meeting it appeared that this decision had been arrived at without consulting the infirmary committees, and the Board very properly decided to refer the matter back in order that their opinions might be obtained. We have always held that the use of a good substitute for butter is a legitimate economy, but the initiative should obviously come from the infirmary committee after consultation with the medical officer, and the recommendation should always include an exact description of the proposed substitute. The Guardians decided at the same time to reduce slightly the weekly allowance of sugar to the staff. This is an economy which has been forced upon many authorities in charge of institutions. Sugar is so important a constituent of a normal diet that its restriction should be decided on only after careful consideration. In the case of most of our institutions a small reduction on the peace-time allowance should be possible without risk of detriment to health, and the limitation of the supply makes any possible economy in use essential.

Some nurses are untidy people—they have not much time to spare for sewing on buttons and mending, so when the matron visits the laundry she is often depressed at the sight of torn and buttonless uniform-dresses, etc. One of our correspondents describes a plan she has employed to encourage habits of neatness in her nurses. On Saturday mornings a fire is lighted in the board-room, and a store of buttons, tapes, cottons, etc., laid out on the table. The night nurses, and those of the day staff who may be off duty in the morning, are invited to bring their laundry and mend it while the matron reads aloud a short story or chats to the nurses as they sew and darn garments which might otherwise have been hurriedly sewn before going to bed, or left to drop to pieces. The fact that the matron cares enough for habits of neatness to make this effort to help her nurses to acquire it has in itself a marked effect, and our correspondent tells us that the alternative of constantly finding fault on account of untidiness is, in the end, more tiring to her than the time and energy spent at the Sewing Bee. Besides, a sister can usually be found to act as reader if matron is called away to other duties.

The South London Hospital for Women, Clapham Common, which the Queen is to open (in May probably), presents features of great interest. It will provide special accommodation for women who, though not poor, are unable to pay the fees demanded at a West-End Nursing Home. There are several private wards to accommodate one or two patients each, and also cubicles. Three ordinary wards are provided for poorer patients. An out-patient department in connection with the hospital has been working for over two years, and will be greatly extended when the hospital is opened. This hospital is one to visit and study. Its board numbers several well-known women, and its chairman is the Marchioness of Londonderry, who has displayed zeal, capacity, and businesslike qualities of a high order.

A story of our King's wonderful memory for faces is told by the sister of an accident ward in one of the great Metropolitan hospitals. His Majesty during a visit to this institution entered into conversation with a patient who had been admitted on account of a fractured femur. Two years later King George V. again visited this hospital, and in passing through the same ward recognised the man to whom he had spoken on his previous visit. "That man," said His Majesty, "was here the last time I came, but he was in that bed over there." "Yus, yer Majesty," said the patient, "it's the other leg this toime."

*** It is our invariable practice to pay for all items of news which we may publish. The minimum payment is 5s. Paragraphs of about twenty lines in length—that is to say, of 150 words or less—are preferred. In this section during the war we propose to give 10s. to the correspondent who may send us in any week the best incident connected with hospital life and work. In this way we hope to afford to all practical aid and encouragement; while those who reciprocate will be able to render us invaluable service. Contributions should be addressed to The Editor, The Hospital, 28 and 29 Southampton Street, Strand, London, W.C., and to be in time for the current week's issue should reach him by the first post on Mondays.

**For Matrons' Appointments see page 62.**

